# 3-[5-(2,4-Dichloro­phen­yl)-1-phenyl-4,5-dihydro-1*H*-pyrazol-3-yl]-4-hy­droxy-2*H*-chromen-2-one

**DOI:** 10.1107/S1600536811001590

**Published:** 2011-01-22

**Authors:** Mohammad Asad, Chuan-Wei Oo, Hasnah Osman, Mohd Mustaqim Rosli, Hoong-Kun Fun

**Affiliations:** a School of Chemical Sciences, Universiti Sains Malaysia, 11800 USM, Penang, Malaysia; b X-ray Crystallography Unit, School of Physics, Universiti Sains Malaysia, 11800 USM, Penang, Malaysia

## Abstract

In the title compound, C_24_H_16_Cl_2_N_2_O_3_, the chromene ring system is almost planar, with a maximum deviation of 0.042 (1) Å. It makes dihedral angles of 3.72 (6), 73.37 (5) and 12.00 (5)° with the dihydro­pyrazole, benzene and phenyl rings, respectively. An intra­molecular O—H⋯N hydrogen bond forms an *S*(6) ring motif. In the crystal, mol­ecules are linked *via* C—H⋯O inter­actions, forming an infinite chain along the *a* axis. The crystal packing is further stabilized by a π–π stacking inter­action [centroid–centroid distance = 3.5471 (7) Å] and a Cl⋯Cl short contact [Cl⋯Cl = 3.214 (1) Å].

## Related literature

For a related structure, see: Asad *et al.* (2010 ▶). For the biological activity of pyrazoline derivatives, see: Bernstein *et al.* (1947 ▶); Chimenti *et al.* (2004 ▶); Goodell *et al.* (2006 ▶); Hollis *et al.* (1984 ▶); Mohammad *et al.* (2008 ▶); Siddiqui *et al.* (2008 ▶). For the stability of the temperature controller used in the data collection, see: Cosier & Glazer (1986 ▶).
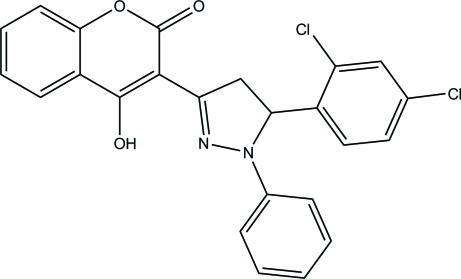


## Experimental

### 

#### Crystal data

C_24_H_16_Cl_2_N_2_O_3_
*M* *_r_* = 451.29
Triclinic, 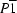

*a* = 6.2583 (2) Å
*b* = 11.5136 (5) Å
*c* = 14.2248 (5) Å
α = 87.242 (1)°
β = 88.821 (1)°
γ = 76.181 (1)°
*V* = 994.11 (6) Å^3^
*Z* = 2
Mo *K*α radiation
μ = 0.36 mm^−1^
*T* = 100 K
0.46 × 0.15 × 0.13 mm

#### Data collection

Bruker SMART APEXII CCD area-detector diffractometer
Absorption correction: multi-scan (*SADABS*; Bruker, 2009 ▶) *T*_min_ = 0.852, *T*_max_ = 0.955
22849 measured reflections
5747 independent reflections
5151 reflections with *I* > 2σ(*I*)
*R*_int_ = 0.020

#### Refinement

*R*[*F* ^2^ > 2σ(*F* ^2^)] = 0.038
*wR*(*F* ^2^) = 0.106
*S* = 1.03
5747 reflections
284 parameters
H atoms treated by a mixture of independent and constrained refinement
Δρ_max_ = 1.11 e Å^−3^
Δρ_min_ = −0.88 e Å^−3^

### 

Data collection: *APEX2* (Bruker, 2009 ▶); cell refinement: *SAINT* (Bruker, 2009 ▶); data reduction: *SAINT*; program(s) used to solve structure: *SHELXTL* (Sheldrick, 2008 ▶); program(s) used to refine structure: *SHELXTL*; molecular graphics: *SHELXTL*; software used to prepare material for publication: *SHELXTL* and *PLATON* (Spek, 2009 ▶).

## Supplementary Material

Crystal structure: contains datablocks global, I. DOI: 10.1107/S1600536811001590/is2660sup1.cif

Structure factors: contains datablocks I. DOI: 10.1107/S1600536811001590/is2660Isup2.hkl

Additional supplementary materials:  crystallographic information; 3D view; checkCIF report

## Figures and Tables

**Table 1:** Hydrogen-bond geometry (Å, °)

*D*—H⋯*A*	*D*—H	H⋯*A*	*D*⋯*A*	*D*—H⋯*A*
O3—H1*O*3⋯N1	0.86 (2)	1.80 (3)	2.5655 (14)	148 (2)
C14—H14*A*⋯O2^i^	0.93	2.40	3.2747 (17)	157

Symmetry code: (i) 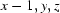.

**Fig. 1.**
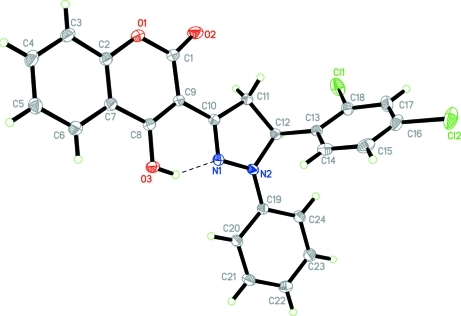
The molecular structure of the title compound, showing 50% probability displacement ellipsoids and the atom-numbering scheme. Hydrogen atoms are shown as spheres of arbitrary radius.

**Fig. 2.**
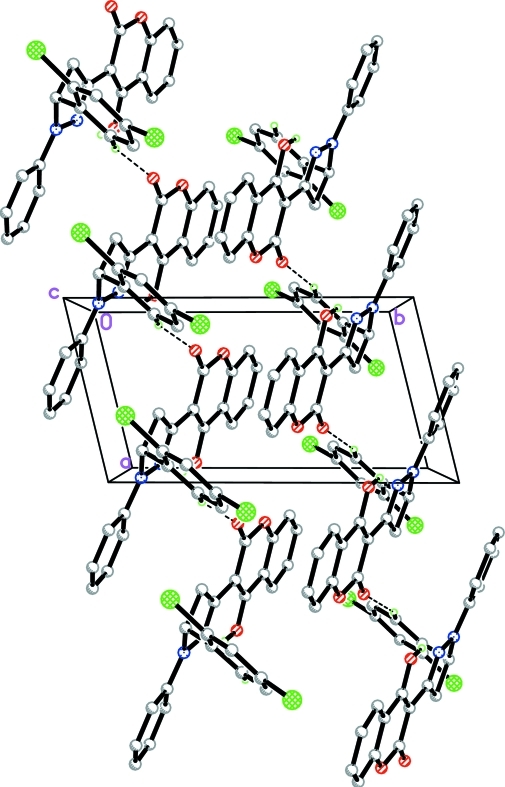
The crystal packing of the title compound viewed along the c axis, showing a molecular chain along the a axis. Dashed lines indicate hydrogen bonds. H atoms not involved in the hydrogen bond interactions have been omitted for clarity.

## supplementary crystallographic information

### Comment

In our earlier work we have reported the crystal structure of chalcone (Asad *et al.*, 2010). In continuation of our work, we reported here the crystal structure of pyrazoline (title compound) which was afforded by the condensation of chalcone with phenylhydrazine. A large number of pyrazoline derivatives showed a broad range of biological properties such as antibacterial (Siddiqui *et al.*, 2008), antiviral (Goodell *et al.*, 2006), antiparasitic (Bernstein *et al.*, 1947), anti-inflammatory (Mohammad *et al.*, 2008), antidepressant (Chimenti *et al.*, 2004) and anticancer (Hollis *et al.*, 1984) activities.

All geometrical parameters of the title compound, (I), are within normal ranges. The chromene group is almost planar with a maximum deviation of 0.042 (1) Å for atom C1. It makes dihedral angles of 3.72 (6), 73.37 (5) and 12.00 (5)° with C10-C12/N1-N1 pyrazole and C13-C18 benzene and C19-C24 phenyl rings, respectively. An intramolecular interaction of O3—H1O3···N1 (Table 1) forms an S(6) hydrogen ring motif.

In the crystal structure, the molecules are linked *via* C14—H14···O2^i^ (Table 1) to form infinite chains along the *a* axis. The crystal packing is further stabilized by π–π stacking interactions with *Cg*–*Cg* distance of 3.5471 (7) Å (*Cg* = centroid of O1/C1-C2/C7-C9) together with Cl1···Cl1 short contact [Cl1···Cl1(1-x, 2-y, -z) = 3.214 (1) Å].

### Experimental

The compound, 3-[(*E*)-3-(2,4-dichlorophenyl)prop-2-enoyl]-4-hydroxy-2H-chromen-2-one (2.76 mmol, 1.00 g) was dissolved in acetic acid (20 ml) and phenylhydrazine (2.76 mmol, 0.30 g) was added to it. The reaction mixture was refluxed on a heating mantle for 2 h. After the reaction was over, the reaction mixture was cooled to room temperature and poured into ice cold water. The yellow-colour solid formed was filtered, washed with water, dried and recrystallized from chloroform-methanol (80:20 *v*/*v*) to get the pure title compound in 62.5% yield.

### Refinement

H1O3 was located in a difference Fourier map and freely refined. The remaining H atoms were positioned geometrically and refined using a riding model (C—H = 0.97 Å for methylene, 0.98 Å for methine and 0.93 Å for the rest of H atoms) with *U*_iso_(H) = 1.2*U*_eq_(C). The highest residual electron density peak is located 0.78 Å from atom Cl2.

### Crystal data

**Table:**

C_24_H_16_Cl_2_N_2_O_3_	*Z* = 2
*M**_r_* = 451.29	*F*(000) = 464
Triclinic, *P*1	*D*_x_ = 1.508 Mg m^−^^3^
Hall symbol: -P 1	Mo *K*α radiation, λ = 0.71073 Å
*a* = 6.2583 (2) Å	Cell parameters from 9965 reflections
*b* = 11.5136 (5) Å	θ = 2.3–32.7°
*c* = 14.2248 (5) Å	µ = 0.36 mm^−^^1^
α = 87.242 (1)°	*T* = 100 K
β = 88.821 (1)°	Block, yellow
γ = 76.181 (1)°	0.46 × 0.15 × 0.13 mm
*V* = 994.11 (6) Å^3^	

### Data collection

**Table:**

Bruker SMART APEXII CCD area-detector diffractometer	5747 independent reflections
Radiation source: fine-focus sealed tube	5151 reflections with *I* > 2σ(*I*)
graphite	*R*_int_ = 0.020
φ and ω scans	θ_max_ = 30.0°, θ_min_ = 1.8°
Absorption correction: multi-scan (*SADABS*; Bruker, 2009)	*h* = −8→8
*T*_min_ = 0.852, *T*_max_ = 0.955	*k* = −16→16
22849 measured reflections	*l* = −20→18

### Refinement

**Table:**

Refinement on *F*^2^	Primary atom site location: structure-invariant direct methods
Least-squares matrix: full	Secondary atom site location: difference Fourier map
*R*[*F*^2^ > 2σ(*F*^2^)] = 0.038	Hydrogen site location: inferred from neighbouring sites
*wR*(*F*^2^) = 0.106	H atoms treated by a mixture of independent and constrained refinement
*S* = 1.03	*w* = 1/[σ^2^(*F*_o_^2^) + (0.0484*P*)^2^ + 0.7425*P*] where *P* = (*F*_o_^2^ + 2*F*_c_^2^)/3
5747 reflections	(Δ/σ)_max_ = 0.001
284 parameters	Δρ_max_ = 1.11 e Å^−^^3^
0 restraints	Δρ_min_ = −0.88 e Å^−^^3^

### Special details

**Table:**

Experimental. The crystal was placed in the cold stream of an Oxford Cyrosystems Cobra open-flow nitrogen cryostat (Cosier & Glazer, 1986) operating at 100.0 (1) K.
Geometry. All esds (except the esd in the dihedral angle between two l.s. planes) are estimated using the full covariance matrix. The cell esds are taken into account individually in the estimation of esds in distances, angles and torsion angles; correlations between esds in cell parameters are only used when they are defined by crystal symmetry. An approximate (isotropic) treatment of cell esds is used for estimating esds involving l.s. planes.
Refinement. Refinement of F^2^ against ALL reflections. The weighted R-factor wR and goodness of fit S are based on F^2^, conventional R-factors R are based on F, with F set to zero for negative F^2^. The threshold expression of F^2^ > 2sigma(F^2^) is used only for calculating R-factors(gt) etc. and is not relevant to the choice of reflections for refinement. R-factors based on F^2^ are statistically about twice as large as those based on F, and R- factors based on ALL data will be even larger.

### Fractional atomic coordinates and isotropic or equivalent isotropic displacement parameters (Å^2^)

**Table:**

	*x*	*y*	*z*	*U*_iso_*/*U*_eq_	
Cl1	0.35949 (7)	0.90740 (4)	0.03853 (2)	0.03009 (10)	
Cl2	−0.14981 (8)	0.62457 (3)	−0.06976 (3)	0.03714 (11)	
O1	0.71612 (15)	0.58755 (9)	0.51861 (7)	0.01973 (19)	
O2	0.72747 (16)	0.66920 (10)	0.37635 (7)	0.0251 (2)	
O3	0.06651 (15)	0.75993 (9)	0.55672 (7)	0.02046 (19)	
N1	0.07093 (17)	0.86134 (9)	0.39234 (7)	0.0162 (2)	
N2	−0.00154 (18)	0.92564 (10)	0.31061 (7)	0.0192 (2)	
C1	0.6146 (2)	0.66136 (11)	0.44534 (9)	0.0177 (2)	
C2	0.6117 (2)	0.57587 (11)	0.60307 (9)	0.0173 (2)	
C3	0.7375 (2)	0.50505 (11)	0.67383 (10)	0.0205 (2)	
H3A	0.8836	0.4664	0.6630	0.025*	
C4	0.6386 (2)	0.49384 (12)	0.76082 (10)	0.0217 (3)	
H4A	0.7202	0.4476	0.8091	0.026*	
C5	0.4182 (2)	0.55092 (12)	0.77721 (9)	0.0218 (3)	
H5A	0.3550	0.5431	0.8362	0.026*	
C6	0.2942 (2)	0.61889 (12)	0.70583 (9)	0.0203 (2)	
H6A	0.1469	0.6555	0.7164	0.024*	
C7	0.3911 (2)	0.63252 (11)	0.61735 (9)	0.0170 (2)	
C8	0.2763 (2)	0.70665 (11)	0.54083 (9)	0.0164 (2)	
C9	0.38412 (19)	0.72235 (11)	0.45724 (8)	0.0156 (2)	
C10	0.2732 (2)	0.80047 (11)	0.38073 (8)	0.0153 (2)	
C11	0.3638 (2)	0.82338 (11)	0.28433 (9)	0.0168 (2)	
H11A	0.4255	0.7491	0.2538	0.020*	
H11B	0.4754	0.8688	0.2875	0.020*	
C12	0.1564 (2)	0.89691 (11)	0.23291 (8)	0.0157 (2)	
H12A	0.1858	0.9707	0.2043	0.019*	
C13	0.07859 (19)	0.82708 (11)	0.15820 (9)	0.0153 (2)	
C14	−0.0757 (2)	0.75932 (11)	0.17822 (10)	0.0199 (2)	
H14A	−0.1327	0.7566	0.2390	0.024*	
C15	−0.1455 (2)	0.69604 (12)	0.10939 (11)	0.0248 (3)	
H15A	−0.2486	0.6514	0.1237	0.030*	
C16	−0.0596 (2)	0.69996 (12)	0.01883 (10)	0.0244 (3)	
C17	0.0968 (2)	0.76382 (12)	−0.00390 (10)	0.0243 (3)	
H17A	0.1558	0.7648	−0.0644	0.029*	
C18	0.1630 (2)	0.82658 (12)	0.06681 (9)	0.0189 (2)	
C19	−0.2083 (2)	1.00293 (10)	0.30513 (8)	0.0154 (2)	
C20	−0.3406 (2)	1.03064 (11)	0.38552 (9)	0.0177 (2)	
H20A	−0.2880	0.9997	0.4444	0.021*	
C21	−0.5506 (2)	1.10448 (12)	0.37687 (9)	0.0201 (2)	
H21A	−0.6383	1.1216	0.4304	0.024*	
C22	−0.6326 (2)	1.15338 (12)	0.28987 (10)	0.0212 (2)	
H22A	−0.7743	1.2018	0.2848	0.025*	
C23	−0.4991 (2)	1.12856 (12)	0.21073 (9)	0.0207 (2)	
H23A	−0.5509	1.1623	0.1524	0.025*	
C24	−0.2890 (2)	1.05397 (11)	0.21745 (9)	0.0186 (2)	
H24A	−0.2017	1.0379	0.1637	0.022*	
H1O3	0.016 (4)	0.804 (2)	0.5079 (18)	0.048 (6)*	

### Atomic displacement parameters (Å^2^)

**Table:**

	*U*^11^	*U*^22^	*U*^33^	*U*^12^	*U*^13^	*U*^23^
Cl1	0.0363 (2)	0.0397 (2)	0.01977 (16)	−0.02068 (16)	0.01019 (13)	−0.00255 (13)
Cl2	0.0479 (2)	0.02154 (17)	0.0407 (2)	−0.00267 (15)	−0.02561 (18)	−0.00634 (14)
O1	0.0146 (4)	0.0231 (4)	0.0190 (4)	0.0003 (3)	−0.0009 (3)	−0.0002 (3)
O2	0.0161 (4)	0.0323 (5)	0.0233 (5)	0.0001 (4)	0.0039 (4)	0.0024 (4)
O3	0.0141 (4)	0.0267 (5)	0.0172 (4)	0.0011 (3)	0.0017 (3)	0.0027 (4)
N1	0.0159 (5)	0.0181 (5)	0.0129 (4)	−0.0009 (4)	−0.0002 (4)	0.0001 (4)
N2	0.0168 (5)	0.0244 (5)	0.0121 (5)	0.0032 (4)	0.0022 (4)	0.0013 (4)
C1	0.0146 (5)	0.0193 (5)	0.0181 (5)	−0.0016 (4)	−0.0010 (4)	−0.0018 (4)
C2	0.0175 (5)	0.0167 (5)	0.0172 (5)	−0.0027 (4)	−0.0019 (4)	−0.0023 (4)
C3	0.0196 (6)	0.0174 (5)	0.0229 (6)	−0.0005 (4)	−0.0054 (5)	−0.0025 (4)
C4	0.0268 (6)	0.0166 (5)	0.0206 (6)	−0.0024 (5)	−0.0073 (5)	0.0002 (4)
C5	0.0267 (6)	0.0199 (6)	0.0178 (6)	−0.0039 (5)	−0.0007 (5)	0.0007 (4)
C6	0.0196 (6)	0.0206 (6)	0.0190 (6)	−0.0022 (5)	0.0004 (4)	0.0011 (4)
C7	0.0169 (5)	0.0166 (5)	0.0168 (5)	−0.0024 (4)	−0.0015 (4)	−0.0008 (4)
C8	0.0144 (5)	0.0168 (5)	0.0172 (5)	−0.0019 (4)	−0.0009 (4)	−0.0016 (4)
C9	0.0132 (5)	0.0175 (5)	0.0153 (5)	−0.0019 (4)	−0.0006 (4)	−0.0021 (4)
C10	0.0140 (5)	0.0170 (5)	0.0147 (5)	−0.0033 (4)	0.0003 (4)	−0.0024 (4)
C11	0.0140 (5)	0.0205 (5)	0.0156 (5)	−0.0035 (4)	0.0008 (4)	−0.0015 (4)
C12	0.0149 (5)	0.0176 (5)	0.0139 (5)	−0.0028 (4)	0.0021 (4)	−0.0011 (4)
C13	0.0135 (5)	0.0155 (5)	0.0157 (5)	−0.0013 (4)	0.0001 (4)	−0.0001 (4)
C14	0.0157 (5)	0.0183 (5)	0.0249 (6)	−0.0031 (4)	0.0022 (5)	0.0005 (5)
C15	0.0178 (6)	0.0176 (6)	0.0392 (8)	−0.0047 (5)	−0.0043 (5)	−0.0005 (5)
C16	0.0274 (7)	0.0161 (5)	0.0281 (7)	−0.0003 (5)	−0.0130 (5)	−0.0035 (5)
C17	0.0325 (7)	0.0220 (6)	0.0167 (6)	−0.0026 (5)	−0.0037 (5)	−0.0017 (5)
C18	0.0207 (6)	0.0201 (6)	0.0162 (5)	−0.0055 (5)	0.0006 (4)	0.0003 (4)
C19	0.0154 (5)	0.0144 (5)	0.0156 (5)	−0.0018 (4)	0.0003 (4)	−0.0015 (4)
C20	0.0188 (6)	0.0183 (5)	0.0148 (5)	−0.0020 (4)	0.0012 (4)	−0.0016 (4)
C21	0.0191 (6)	0.0199 (6)	0.0197 (6)	−0.0011 (5)	0.0041 (4)	−0.0029 (4)
C22	0.0176 (6)	0.0195 (6)	0.0240 (6)	0.0010 (4)	0.0001 (5)	−0.0018 (5)
C23	0.0218 (6)	0.0192 (6)	0.0184 (6)	0.0003 (5)	−0.0024 (5)	0.0007 (4)
C24	0.0202 (6)	0.0178 (5)	0.0157 (5)	−0.0005 (4)	0.0013 (4)	−0.0001 (4)

### Geometric parameters (Å, °)

**Table:**

Cl1—C18	1.7408 (14)	C11—C12	1.5464 (17)
Cl2—C16	1.7402 (14)	C11—H11A	0.9700
O1—C2	1.3720 (16)	C11—H11B	0.9700
O1—C1	1.3770 (15)	C12—C13	1.5175 (17)
O2—C1	1.2097 (16)	C12—H12A	0.9800
O3—C8	1.3294 (15)	C13—C18	1.3929 (17)
O3—H1O3	0.86 (3)	C13—C14	1.3958 (17)
N1—C10	1.3037 (16)	C14—C15	1.385 (2)
N1—N2	1.3726 (14)	C14—H14A	0.9300
N2—C19	1.3857 (15)	C15—C16	1.388 (2)
N2—C12	1.4634 (15)	C15—H15A	0.9300
C1—C9	1.4560 (17)	C16—C17	1.383 (2)
C2—C3	1.3944 (17)	C17—C18	1.3911 (18)
C2—C7	1.3946 (17)	C17—H17A	0.9300
C3—C4	1.386 (2)	C19—C20	1.4019 (17)
C3—H3A	0.9300	C19—C24	1.4033 (17)
C4—C5	1.399 (2)	C20—C21	1.3884 (18)
C4—H4A	0.9300	C20—H20A	0.9300
C5—C6	1.3828 (18)	C21—C22	1.3904 (19)
C5—H5A	0.9300	C21—H21A	0.9300
C6—C7	1.4041 (18)	C22—C23	1.3876 (19)
C6—H6A	0.9300	C22—H22A	0.9300
C7—C8	1.4420 (17)	C23—C24	1.3907 (18)
C8—C9	1.3807 (17)	C23—H23A	0.9300
C9—C10	1.4540 (17)	C24—H24A	0.9300
C10—C11	1.5085 (17)		
			
C2—O1—C1	122.30 (10)	N2—C12—C13	112.71 (10)
C8—O3—H1O3	108.6 (17)	N2—C12—C11	102.00 (9)
C10—N1—N2	109.48 (10)	C13—C12—C11	112.55 (10)
N1—N2—C19	121.38 (10)	N2—C12—H12A	109.8
N1—N2—C12	112.49 (10)	C13—C12—H12A	109.8
C19—N2—C12	126.03 (10)	C11—C12—H12A	109.8
O2—C1—O1	116.12 (11)	C18—C13—C14	117.41 (12)
O2—C1—C9	125.99 (12)	C18—C13—C12	120.73 (11)
O1—C1—C9	117.88 (11)	C14—C13—C12	121.85 (11)
O1—C2—C3	116.69 (11)	C15—C14—C13	121.30 (13)
O1—C2—C7	121.53 (11)	C15—C14—H14A	119.4
C3—C2—C7	121.78 (12)	C13—C14—H14A	119.4
C4—C3—C2	118.17 (12)	C14—C15—C16	119.24 (13)
C4—C3—H3A	120.9	C14—C15—H15A	120.4
C2—C3—H3A	120.9	C16—C15—H15A	120.4
C3—C4—C5	121.11 (12)	C17—C16—C15	121.55 (12)
C3—C4—H4A	119.4	C17—C16—Cl2	118.24 (12)
C5—C4—H4A	119.4	C15—C16—Cl2	120.21 (11)
C6—C5—C4	120.11 (13)	C16—C17—C18	117.73 (13)
C6—C5—H5A	119.9	C16—C17—H17A	121.1
C4—C5—H5A	119.9	C18—C17—H17A	121.1
C5—C6—C7	119.86 (12)	C17—C18—C13	122.74 (12)
C5—C6—H6A	120.1	C17—C18—Cl1	117.93 (10)
C7—C6—H6A	120.1	C13—C18—Cl1	119.33 (10)
C2—C7—C6	118.95 (11)	N2—C19—C20	121.34 (11)
C2—C7—C8	117.75 (11)	N2—C19—C24	119.68 (11)
C6—C7—C8	123.25 (11)	C20—C19—C24	118.98 (11)
O3—C8—C9	122.99 (11)	C21—C20—C19	119.77 (12)
O3—C8—C7	116.36 (11)	C21—C20—H20A	120.1
C9—C8—C7	120.63 (11)	C19—C20—H20A	120.1
C8—C9—C10	121.34 (11)	C20—C21—C22	121.39 (12)
C8—C9—C1	119.77 (11)	C20—C21—H21A	119.3
C10—C9—C1	118.88 (11)	C22—C21—H21A	119.3
N1—C10—C9	119.64 (11)	C23—C22—C21	118.75 (12)
N1—C10—C11	112.43 (10)	C23—C22—H22A	120.6
C9—C10—C11	127.92 (11)	C21—C22—H22A	120.6
C10—C11—C12	102.04 (9)	C22—C23—C24	120.94 (12)
C10—C11—H11A	111.4	C22—C23—H23A	119.5
C12—C11—H11A	111.4	C24—C23—H23A	119.5
C10—C11—H11B	111.4	C23—C24—C19	120.14 (12)
C12—C11—H11B	111.4	C23—C24—H24A	119.9
H11A—C11—H11B	109.2	C19—C24—H24A	119.9
			
C10—N1—N2—C19	−176.79 (11)	C9—C10—C11—C12	171.31 (12)
C10—N1—N2—C12	6.61 (15)	N1—N2—C12—C13	109.17 (12)
C2—O1—C1—O2	176.03 (12)	C19—N2—C12—C13	−67.25 (16)
C2—O1—C1—C9	−4.01 (17)	N1—N2—C12—C11	−11.76 (13)
C1—O1—C2—C3	−175.51 (11)	C19—N2—C12—C11	171.82 (12)
C1—O1—C2—C7	4.23 (18)	C10—C11—C12—N2	11.62 (12)
O1—C2—C3—C4	178.39 (11)	C10—C11—C12—C13	−109.42 (11)
C7—C2—C3—C4	−1.36 (19)	N2—C12—C13—C18	157.95 (11)
C2—C3—C4—C5	0.6 (2)	C11—C12—C13—C18	−87.35 (14)
C3—C4—C5—C6	0.7 (2)	N2—C12—C13—C14	−23.34 (16)
C4—C5—C6—C7	−1.3 (2)	C11—C12—C13—C14	91.36 (14)
O1—C2—C7—C6	−178.93 (12)	C18—C13—C14—C15	−1.30 (19)
C3—C2—C7—C6	0.81 (19)	C12—C13—C14—C15	179.95 (11)
O1—C2—C7—C8	−1.45 (18)	C13—C14—C15—C16	0.2 (2)
C3—C2—C7—C8	178.28 (11)	C14—C15—C16—C17	1.2 (2)
C5—C6—C7—C2	0.53 (19)	C14—C15—C16—Cl2	−178.28 (10)
C5—C6—C7—C8	−176.79 (12)	C15—C16—C17—C18	−1.3 (2)
C2—C7—C8—O3	−179.67 (11)	Cl2—C16—C17—C18	178.16 (10)
C6—C7—C8—O3	−2.31 (18)	C16—C17—C18—C13	0.1 (2)
C2—C7—C8—C9	−1.31 (18)	C16—C17—C18—Cl1	−179.87 (10)
C6—C7—C8—C9	176.04 (12)	C14—C13—C18—C17	1.17 (19)
O3—C8—C9—C10	0.68 (19)	C12—C13—C18—C17	179.93 (12)
C7—C8—C9—C10	−177.57 (11)	C14—C13—C18—Cl1	−178.87 (9)
O3—C8—C9—C1	179.66 (11)	C12—C13—C18—Cl1	−0.10 (16)
C7—C8—C9—C1	1.41 (18)	N1—N2—C19—C20	8.45 (19)
O2—C1—C9—C8	−178.87 (13)	C12—N2—C19—C20	−175.43 (12)
O1—C1—C9—C8	1.18 (18)	N1—N2—C19—C24	−170.94 (11)
O2—C1—C9—C10	0.1 (2)	C12—N2—C19—C24	5.18 (19)
O1—C1—C9—C10	−179.82 (11)	N2—C19—C20—C21	−177.32 (12)
N2—N1—C10—C9	−178.26 (11)	C24—C19—C20—C21	2.08 (18)
N2—N1—C10—C11	2.11 (14)	C19—C20—C21—C22	−0.9 (2)
C8—C9—C10—N1	3.12 (18)	C20—C21—C22—C23	−0.9 (2)
C1—C9—C10—N1	−175.87 (11)	C21—C22—C23—C24	1.5 (2)
C8—C9—C10—C11	−177.31 (12)	C22—C23—C24—C19	−0.3 (2)
C1—C9—C10—C11	3.70 (19)	N2—C19—C24—C23	177.91 (12)
N1—C10—C11—C12	−9.10 (13)	C20—C19—C24—C23	−1.49 (19)

### Hydrogen-bond geometry (Å, °)

**Table:**

*D*—H···*A*	*D*—H	H···*A*	*D*···*A*	*D*—H···*A*
O3—H1O3···N1	0.86 (2)	1.80 (3)	2.5655 (14)	148 (2)
C14—H14A···O2^i^	0.93	2.40	3.2747 (17)	157

Symmetry codes: (i) *x*−1, *y*, *z*.
